# Long-Term Epidemiological Trends of Human Adenovirus Infection in South Korea: A Single-Center Study (2007–2024)

**DOI:** 10.3390/pathogens14111143

**Published:** 2025-11-11

**Authors:** Yu Jeong Kim, Sung Hun Jang, Jeong Su Han, Jae-Sik Jeon, Jae Kyung Kim

**Affiliations:** 1 Department of Biomedical Laboratory Science, College of Health Sciences, Dankook University, Cheonan-si 31116, Republic of Korea; bkh04117@naver.com (Y.J.K.); jshan1162@naver.com (J.S.H.); zenty87@naver.com (J.-S.J.); 2 Department of Medical Laser, Graduate School of Medicine, Dankook University, Cheonan-si 31116, Republic of Korea; well8143@naver.com; 3 Research Center for Bio-Functional and Biocompatible Materials, Dankook University, Cheonan-si 31116, Republic of Korea

**Keywords:** age distribution, COVID-19 pandemic, epidemiology, human adenovirus, respiratory virus, seasonality, SDG 3, surveillance

## Abstract

Human adenoviruses (HAdVs; *genus Mastadenovirus*, *family Adenoviridae*) are major etiologic agents of respiratory infections, exerting a disproportionately large impact on children. However, no long-term study to date has spanned pre- and post-pandemic periods in a Korean tertiary setting. Here, we retrospectively analyzed 23,284 nasopharyngeal swabs collected between 2007 and 2024 at a tertiary medical center in South Korea. Most specimens were obtained from inpatients and outpatients presenting with fever or respiratory symptoms for differential viral diagnosis. HAdV was detected using real-time PCR, and positivity rates were compared by year, season, and age group. Overall, 2043 tested positive for HAdV. Annual positivity peaked in 2010, 2013, and 2016, followed by a sharp decline during the coronavirus disease 2019 pandemic, reaching its lowest level in 2024. Positivity was higher in summer and spring than in winter. Children aged 1–5 years had the highest positivity, whereas adults and older adults showed markedly lower rates. The disproportionate burden among children underscores the need for targeted surveillance, school-based infection control, and region-specific preparedness strategies. These findings provide crucial long-term evidence into adenovirus circulation in the post-pandemic era and provides an evidence-based foundation for future public health policy and infection control planning.

## 1. Introduction

Human adenoviruses (HAdVs) are non-enveloped, double-stranded DNA viruses that cause a broad spectrum of clinical manifestations, ranging from mild upper respiratory tract infections to severe pneumonia and disseminated disease. Severe outcomes are particularly common among vulnerable populations such as children, immunocompromised patients, and older adults, with HAdV recognized as a major cause of community-acquired pneumonia in pediatric cohorts [1,2,3,4]. Outbreaks of severe adenovirus infections have been reported in China, Japan, and South Korea as well as globally, underscoring their public health significance [5,6,7].

Previous epidemiological studies have indicated that HAdV circulation often follows cyclical epidemic peaks driven by genotype shifts and the accumulation of susceptible hosts [8,9,10]. Seasonal variation also plays a critical role, with higher detection rates typically observed in warmer months, although localized outbreaks in atypical periods, including winter surges, have been documented in community and institutional settings, suggesting that HAdV transmission dynamics are more complex than a simple seasonal pattern [11,12,13,14].

The coronavirus disease 2019 (COVID-19) pandemic profoundly altered the circulation of respiratory viruses worldwide. Non-pharmaceutical interventions (NPIs), including mask use, school closures, and mobility restrictions, led to unprecedented declines in viral activity, including adenoviruses [15,16,17]. However, unlike influenza and respiratory syncytial virus (RSV), which resurged rapidly following the relaxation of NPIs, adenovirus activity has remained suppressed in several regions [6,18]. This prolonged suppression highlights the urgent need for long-term, region-specific studies to clarify whether HAdV transmission dynamics have fundamentally shifted in the post-pandemic era.

Despite HAdV’s clinical and public health relevance, no long-term study to date has spanned pre- and post-pandemic periods in a Korean tertiary setting. Most previous studies have been restricted to short surveillance windows or focused primarily on pediatric populations [3,19,20]. Consequently, the long-term temporal trends, seasonal characteristics, and age-specific risks of HAdV infection over the past 20 years remain insufficiently understood, representing a major gap in longitudinal data that limits clinical management and development of effective national surveillance strategies.

To address this gap, we conducted an 18-year retrospective analysis of HAdV epidemiology using a large dataset of respiratory specimens collected at a single tertiary medical center in South Korea between 2007 and 2024. This study aimed to: (1) characterize long-term temporal and seasonal trends in HAdV activity, (2) identify age-specific high-risk groups, and (3) evaluate the impact of the COVID-19 pandemic on adenovirus circulation. Our findings provide new insights into the dynamics of HAdV in the post-pandemic era and offer valuable evidence to guide clinical management and strengthen public health preparedness strategies.

## 2. Materials and Methods

### 2.1. Data Collection

This study was approved by the Institutional Review Board (IRB) of Dankook University, Cheonan, South Korea (IRB approval number: DKU 2025-02-004-003), and all procedures were conducted in accordance with the ethical principles outlined in the Declaration of Helsinki. As the study used fully anonymized, retrospective data without any identifiable information, the requirement for informed consent was waived.

HAdV testing data were obtained from Dankook University Hospital, a single tertiary care center in South Korea, spanning the period from 2007 to 2024. The dataset included test date, test result (positive or negative), and patient age, comprising 23,284 specimens. Testing was primarily performed on outpatients and inpatients presenting with influenza-like illness (e.g., fever, cough, sore throat) or requiring differential diagnosis for respiratory infections. Specimens lacking essential demographic information were excluded from the analysis.

Only nasopharyngeal swab specimens obtained for the diagnosis of respiratory infections were included, and cases with non-respiratory tract diseases were not analyzed.

### 2.2. Data Analysis

Following the E11 guidelines of the International Council for Harmonization, study participants were stratified into five age groups: infants (0 years), infancy (1–5 years), kindergarten age (6–8 years), elementary school age (9–12 years), adolescents (13–18 years), adults (19–64 years), and older adults (≥65 years). This classification aligns with age categories commonly used in clinical and epidemiological research and was applied consistently throughout this study.

Among the total specimens, the children group represented the largest proportion (45.3%), followed by older adults (20.2%) and infants (19.5%) (Table 1).

Seasons were categorized as spring (March–May), summer (June–August), autumn (September–November), and winter (December–February), based on the test date. Annual trends were assessed using graphical visualization and relevant statistical metrics.

### 2.3. DNA Extraction and Real-Time PCR

Nasopharyngeal swab specimens were either processed immediately or stored at 4 °C and tested within 24 h if immediate processing was not possible. Viral DNA was extracted using the QIAamp Viral DNA Mini Kit (Qiagen, Hilden, Germany) in accordance with the manufacturer’s instructions.

The extracted DNA was analyzed by real-time Polymerase Chain Reaction (PCR) using a commercial respiratory virus detection kit (LG Life Sciences, Seoul, Republic of Korea). HAdV DNA was detected with virus-specific TaqMan probes and primers provided in the kit. PCR amplification was performed on the multiplex real-Time PCR System (LG Life Sciences, Seoul, Republic of Korea), and all amplification and analysis procedures were performed strictly following the manufacturer’s protocol. From 2007 to 2012, respiratory viruses were detected using the Seeplex RV series multiplex PCR assays (Seegene, Seoul, Republic of Korea), which employed conventional PCR followed by gel electrophoresis, strictly performed according to the manufacturer’s instructions. Since 2013, the laboratory has adopted the AdvanSure RV and RV-Plus real-time RT-PCR kits (LG Life Sciences, Seoul, Republic of Korea) together with the SLAN real-time PCR system (LG Life Sciences, Seoul, Republic of Korea) for multiplex respiratory virus testing, all procedures being conducted in strict accordance with the manufacturer’s protocol. The AdvanSure RV assay targets fourteen respiratory pathogens, and adenovirus detection has been consistently available since 2013, with all analyses performed under the manufacturer’s recommended conditions. All adenovirus-positive results obtained between 2007 and 2024 were included in this analysis, acknowledging that the earlier (2007–2012) data were generated using a different multiplex PCR platform but still following the manufacturer’s guidelines at the time.

### 2.4. Statistical Analysis

All statistical analyses were performed using R software version 4.5.1 (The R Foundation for Statistical Computing, Auckland, New Zealand). The primary outcome variable was HAdV positivity (positive vs. negative), and most independent variables—including age group (infants, children, adolescents, adults, older adults) and season (spring, summer, autumn, winter)—were categorical. Accordingly, chi-square tests were applied to assess associations between these variables. For age- and season-specific comparisons, expected values were calculated by multiplying the overall HAdV positivity rate (8.78%) by the number of specimens in each age group. A significance threshold of *p* < 0.05 was adopted, and all tests were two-tailed. Missing data were excluded using listwise deletion. As most variables were categorical and the study design was retrospective at the population level, no multivariate modeling was performed. Instead, stratified analyses and deviations between observed and expected values were used to identify high-risk groups.

During the preparation of this manuscript, the authors used ChatGPT-5 (OpenAI, San Francisco, CA, USA) solely for English language refinement and style improvement. The authors thoroughly reviewed and verified all AI-assisted text to ensure accuracy, consistency, and scientific integrity. No AI tools were used for data analysis, interpretation, or generation of scientific content.

## 3. Results

### 3.1. Annual HAdV Positivity Trend (2007–2024)

Analysis of the annual number of HAdV-positive cases demonstrated substantial interannual variation over the 18-year study period (χ^2^ = 602.61, df = 17, *p* < 0.001). From 2007 to 2009, the annual number of positive cases ranged from 73 to 127 (positivity rate: 4.85–10.03%), followed by a sharp increase in 2010 (278 cases, 16.77%), which represented the highest annual detection during the entire study period. Between 2011 and 2014, the case numbers fluctuated markedly, ranging from 140 to 260 (positivity rate: 8.66–16.82%), with secondary peaks observed in 2013 (260 cases, 16.82%) and 2016 (195 cases, 11.85%) (Table S1).

From 2017 onward, a gradual decline in HAdV detections was observed, with 148 cases in 2018 (8.06%) and 87 cases in 2019 (6.07%). Beginning in 2020, case numbers dropped sharply, with only 11–22 cases reported annually between 2020 and 2023 (positivity rate: 1.51–2.16%), except for a modest increase in 2023 (22 cases, 2.16%). The lowest detection was recorded in 2024, with only three cases (0.45%).

**Figure 1:**
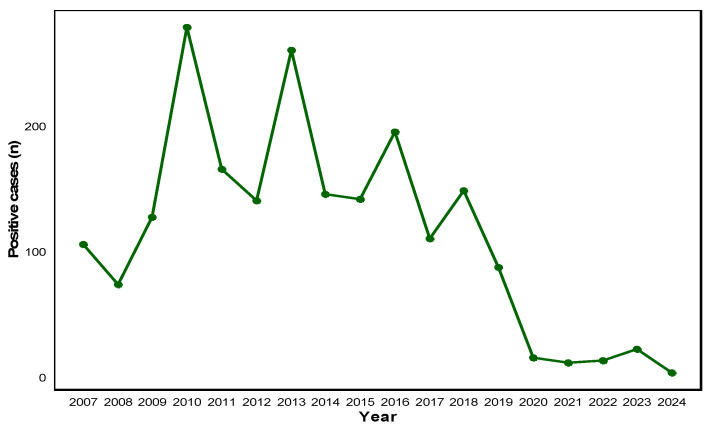
Annual distribution of adenovirus-positive cases from 2007 to 2024. The line graph depicts the yearly number of laboratory-confirmed adenovirus-positive cases recorded between 2007 and 2024. A marked increase was observed during 2010–2013, followed by a gradual decline and sharp decrease during the COVID-19 pandemic period (2020–2022). The data indicate a sustained low level of detection in the post-pandemic years.

Overall, HAdV activity remained consistently high until 2019, after which, it exhibited a marked and sustained decline during the post-pandemic period, with no evidence of recovery to pre-pandemic levels as of 2024 (Figure 1).

### 3.2. Seasonal HAdV Positivity Rate

**Figure 2:**
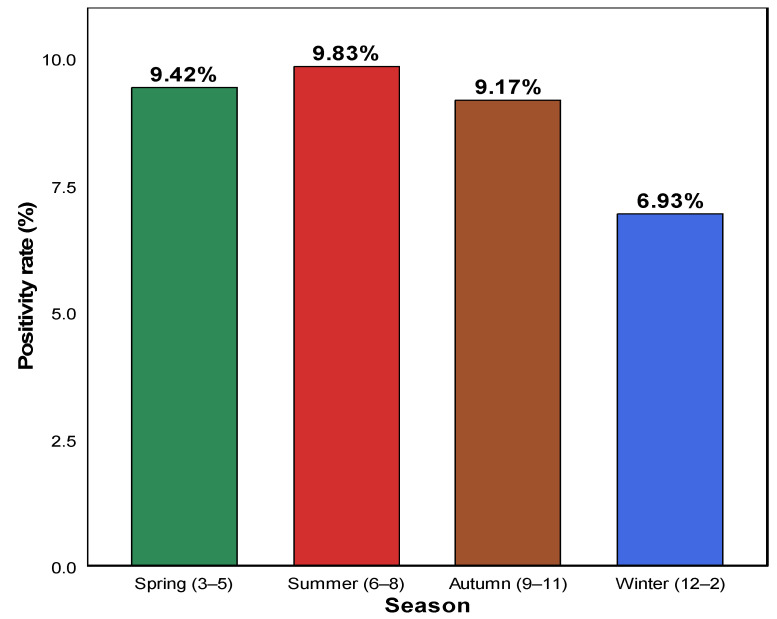
Seasonal positivity rates of adenovirus infections from 2007 to 2024. The bar chart illustrates the seasonal distribution of adenovirus positivity rates based on PCR-confirmed cases. The highest rate was observed in summer (9.83%), followed by spring (9.42%) and autumn (9.17%). Winter showed the lowest positivity rate (6.93%). These findings suggest a predominance of adenovirus activity during the warmer months.

Figure 2 presents the seasonal distribution of HAdV positivity rates based on respiratory specimens collected at a tertiary medical center in South Korea. Seasonal analysis showed that the detection rate was the highest in summer (June–August, 9.83%; χ^2^ = 38.64, df = 3, *p* < 0.001), followed by spring (March–May, 9.42%) and autumn (September–November, 9.17%). In contrast, the positivity rate in winter (December–February) was considerably lower at 6.93% (Table 2; Figure 2).

These findings indicate that the seasonal distribution of HAdV was not random, with relatively higher activity in summer and spring and reduced detection in winter.

### 3.3. HAdV Positivity Rate by Age Group

**Figure 3:**
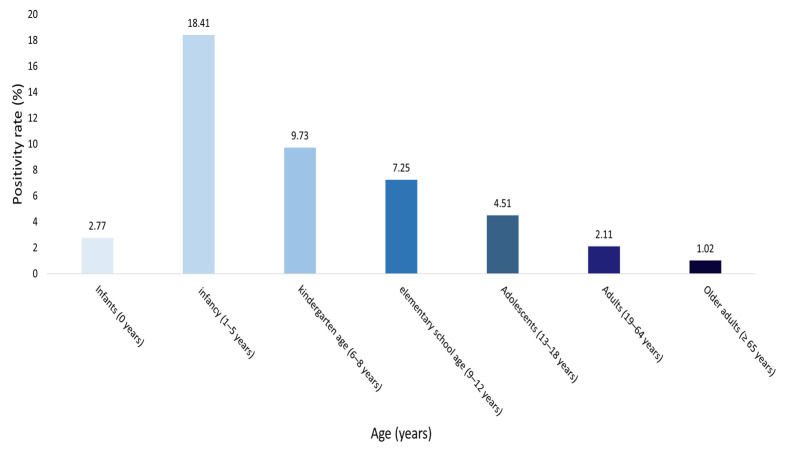
Age-specific positivity rates of adenovirus infections (2007–2024). The bar chart displays the proportion of HAdV-positive cases within each age group. The *x*-axis indicates seven age categories (infants, 1–5 years, 6–8 years, 9–12 years, 13–18 years, 19–64 years, and ≥65 years), and the *y*-axis represents the corresponding positivity rate (%). Bar colors deepen progressively with increasing age. Numerical labels above each bar indicate the exact positivity percentage for each group.

Among the 23,284 individuals tested, adenovirus (HAdV) positivity rates differed significantly across age groups (χ^2^ = 1776.5, df = 6, *p* < 0.001; Table S2). Detailed age-stratified analysis revealed that the highest positivity rate was observed in children aged 1–5 years (18.4%), followed by those aged 6–8 years (9.7%), 9–12 years (7.3%), 13–18 years (4.3%), and infants aged 0 years (2.8%). In contrast, adults (19–64 years, 2.1%) and older adults (≥65 years, 1.0%) exhibited markedly lower positivity rates (Table 3). These findings demonstrate that HAdV susceptibility was disproportionately concentrated in children aged 1–5 years and declined sharply with increasing age (Figure 3).

## 4. Discussion

This 18-year retrospective study, encompassing 23,284 respiratory specimens from a single tertiary medical center, provides valuable long-term insights into the epidemiology of HAdV infection in South Korea. Recurrent epidemic peaks were observed before the COVID-19 pandemic, followed by a sharp decline during the pandemic period and sustained suppression through 2024. HAdV showed the expected spring–summer predominance, and a particularly high burden was observed among children. In contrast, older adults exhibited a markedly lower positivity rate, suggesting differences in exposure frequency or immune protection across age groups.

Annual analysis identified distinct epidemic peaks in 2010, 2013, and 2016, consistent with the cyclical nature of adenovirus activity previously reported in Asian countries [5,8,10]. These periodic outbreaks are likely driven by genotype shifts and the accumulation of susceptible hosts [9,21]. Since 2020, the implementation of NPIs—including mask use, hand hygiene, and social distancing—has led to a substantial decline in HAdV detections, paralleling reductions in respiratory virus transmission reported in Brazil, China, South Africa, and Hong Kong [13,15,16,17]. Similarly, nationwide surveillance data from South Korea also reported a sharp decline in adenovirus activity after 2020, showing a distribution pattern comparable to that observed in the present study [22]. This concordance supports the possibility that the findings of this single-center study are representative within the broader national epidemiological context. However, unlike influenza and RSV, which rapidly resurged following the relaxation of NPIs, adenovirus activity remained markedly suppressed through 2024, indicating distinct ecological dynamics [6,18]. This sustained suppression may indicate not only ecological but also behavioral adaptation, warranting integration of adenovirus monitoring into national respiratory virus surveillance frameworks. This sustained suppression, despite the normalization of social behaviors, suggests a post-pandemic adenovirus circulation shift—a delayed ecological recovery and restructured transmission equilibrium distinct from other respiratory viruses. For influenza and RSV, the concept of “immunity debt” has been proposed, wherein reduced viral exposure during the pandemic resulted in temporary immune gaps and subsequent rapid rebounds [23,24]. This concept has been extensively discussed for RSV and influenza but remains less explored for adenovirus, highlighting the need for mechanistic studies addressing its post-pandemic transmission dynamics. In contrast, adenovirus appears less influenced by short-term immunity gaps and may instead exhibit delayed reactivation, as observed in Japan and Chile [7,21]. These findings underscore the importance of continued surveillance even after the resurgence of other respiratory pathogens.

Seasonal analysis revealed that HAdV activity was the highest in summer, followed by spring and autumn, and the lowest in winter. This seasonal pattern suggests that HAdV transmission in South Korea follows a spring–summer predominance rather than the winter peak typically observed for influenza and RSV. Consistently, previous studies in South Korea have also reported increased adenovirus circulation during the summer months, while similar trends of heightened activity during warmer seasons have been documented in neighboring East Asian countries, including China [22,25]. The summer predominance may be associated with environmental and behavioral factors that facilitate viral transmission. High temperature and humidity can enhance viral stability in aerosols and on surfaces, while increased social contact in schools and childcare facilities during this period may further promote transmission [11,14,26,27,28,29]. These findings provide important insights into the ecological characteristics of adenovirus circulation in South Korea and underscore the need for seasonally tailored infection-control strategies. Strengthening preventive measures during the spring and summer seasons may help to mitigate the spread of adenovirus infections within community and healthcare settings.

Age-specific analysis revealed that the burden of HAdV infection was disproportionately high among children aged 1–5 years, with a positivity rate (18.4%) exceeding more than twice the overall mean, consistent with trends reported in international surveillance data [3,7,19]. Severe pediatric infections frequently required intensive care and were associated with considerable mortality [1,2,4]. Among all age groups, children aged 1–5 years and older adults aged ≥65 years exhibited the largest deviations from the expected distribution. The low positivity observed in older adults may reflect reduced exposure opportunities, cross-protection from prior infections, or a relatively lower rate of diagnostic testing in this population [18,20]. These findings underscore the need for future multicenter studies to provide a more comprehensive understanding of age-related differences in adenovirus epidemiology.

This study has few limitations. First, different adenovirus types are known to exhibit distinct clinical manifestations and disease severities; however, type-specific data were unavailable in this study, precluding genotype-based epidemiological or clinical comparisons. In Korea and other Asian countries, HAdV-B3 and HAdV-E4 have been identified as major causes of large pediatric outbreaks, whereas HAdV-C2 has been frequently detected in surveillance studies [30,31]. Therefore, it remains uncertain whether the observed periodic fluctuations or the spring–summer peak was associated with shifts in circulating genotypes. Future research incorporating molecular typing analyses is warranted to elucidate genotype-specific epidemiological and clinical characteristics. Second, because the dataset was derived from laboratory testing records, only the presence of respiratory symptoms at the time of sampling was available. Detailed clinical classification by year—such as pneumonia, bronchitis, or upper respiratory infection—was not recorded, which limits the clinical interpretability of the findings. Additionally, because detailed clinical outcome data such as disease severity, ICU admission, or mortality were not included in the laboratory dataset, it was not possible to evaluate temporal trends in severe adenovirus infections. Future studies linking laboratory results with clinical records are warranted to better understand the burden and progression of severe cases. Third, the single-center retrospective design limits the generalizability of the results. Fourth, although all tests were conducted under standardized laboratory protocols, advances in diagnostic technology and variations in testing volume over the 18-year period may have influenced the observed positivity trends. Moreover, because detailed clinical data on patients’ immune status (e.g., history of transplantation, malignancy, or immunosuppressive therapy) were not available, it was not feasible to distinguish between immunocompetent and immunocompromised hosts. Future large-scale studies integrating clinical metadata are warranted to clarify immune status–specific patterns and outcomes of adenovirus infection.

Future research should include nationwide surveillance incorporating molecular typing to clarify genotype dynamics and elucidate the mechanisms underlying the spring–summer peaks and the low positivity observed among older adults. Moreover, large-scale studies integrating individual epidemiological, immunological (including immune deficiency status), and clinical data are needed to further refine risk stratification and infection control strategies. In addition, future investigations should integrate multi-virus surveillance data to evaluate co-infection patterns and their potential impact on adenovirus transmission dynamics.

Despite these limitations, this study provides robust long-term evidence on the epidemiology of adenovirus in South Korea. Based on the observed spring–summer predominance and pandemic-associated suppression, these findings may inform the design of future vaccination and surveillance strategies, as well as reinforce infection control measures in schools and childcare facilities. Strengthening hygiene education, particularly regular handwashing among children, and implementing targeted public health interventions could serve as key components of effective adenovirus prevention and preparedness policies.

## 5. Conclusions

This 18-year longitudinal study elucidated the epidemiology of human adenovirus infections in South Korea. The pronounced suppression during the pandemic and the consistent spring–summer predominance highlights the distinct ecological and seasonal characteristics of adenovirus circulation. These findings reinforce infection control in schools and childcare facilities and support targeted hygiene education and public health interventions for children.

## Figures and Tables

**Table 1:** Distribution of the study participants (*n* = 23,284) by age, group, and sex.

Age Group	Total Individuals (*n*)	Male (*n*)	Female (*n*)	Percentage (%)
Infants (0 years)	4543	2704	1839	19.5
Infancy (1–5 years)	8886	5055	3831	38.2
Kindergarten age (6–8 years)	976	555	421	4.2
Elementary school age (9–12 years)	675	382	293	2.8
Adolescents (13–18 years)	577	336	241	2.5
Adults (19–64 years)	2935	1893	1042	12.6
Older adults (≥65 years)	4692	3036	1656	20.2
Total	23,284	13,961	9323	100

**Table 2:** Seasonal distribution of adenovirus-positive cases and positivity rates (2007–2024).

Season	Total Individuals (*n*)	Positive Case (*n*)	Positivity Rate (%)
Spring (3–5)	6391	602	9.42%
Summer (6–8)	4810	473	9.83%
Autumn (9–11)	5607	514	9.17%
Winter (12–2)	6476	449	6.93%

**Table 3:** Adenovirus positivity rates by age group from 2007 to 2024.

Age Group	Total Individuals (*n*)	Positive (*n*)	Negative (*n*)	Positivity Rate (%)
Infants (0 years)	4543	125	4418	2.77
Infancy (1–5 years)	8886	1636	7250	18.41
Kindergarten age (6–8 years)	976	95	881	9.73
Elementary school age (9–12 years)	675	49	626	7.25
Adolescents (13–18 years)	577	25	552	4.51
Adults (19–64 years)	2935	61	2874	2.11
Older adults (≥ 65 years)	4692	47	4645	1.02

## Data Availability

The data that support the findings of this study are derived from patient records at Dankook University Hospital and are subject to ethical and legal restrictions. Due to privacy and confidentiality concerns, the raw datasets cannot be made publicly available. However, anonymized summary data are available from the corresponding author upon reasonable request, subject to approval by the Institutional Review Board.

## Supplementary Materials

The following supporting information can be downloaded at: https://www.mdpi.com/article/10.3390/pathogens14111143/s1, Table S1: Annual distribution of adenovirus testing results and positivity rates from 2007 to 2024; Table S2: Adenovirus-positive observed and expected chi-square contributions by age group from 2007 to 2024.

## Abbreviations

The following abbreviations are used in this manuscript:

HAdVs	Human adenoviruses
COVID-19	Coronavirus disease 2019
NPI	Non-pharmaceutical interventions
RSV	respiratory syncytial virus
