# Supplementary material for: Long-Term Epidemiological Trends of Human Adenovirus Infection in South Korea: A Single-Center Study (2007–2024)
Source: Pathogens. 2025 Nov 11;14(11):1143. doi: 10.3390/pathogens14111143 (PMC12655292; doi:10.3390/pathogens14111143)
Supplement: Supplementary file 1 [file pathogens-14-01143-s001.zip › pathogens-3959129-supplementary.pdf]

Supplementary Materials

Table S1. Annual distribution of adenovirus testing results and positivity rates from 2007 to 2024.

Year	Total (n)	Positive Case (n)	Negative Case (n)	Positivity Rate (%)
2007	1057	105	952	9.93
2008	1504	73	1431	4.85
2009	1265	127	1138	10.03
2010	1657	278	1379	16.77
2011	1568	165	1403	10.52
2012	1345	140	1205	10.40
2013	1545	260	1285	16.82
2014	1674	145	1529	8.66
2015	1388	141	1247	10.15
2016	1645	195	1450	11.85
2017	1436	110	1326	7.66
2018	1834	148	1686	8.06
2019	1432	87	1345	6.07
2020	792	15	777	1.89
2021	613	11	602	1.79
2022	860	13	847	1.51
2023	1016	22	994	2.16
2024	653	3	650	0.45

Table S2. Adeno virus positive observed and expected chi-square contributions by age group from 2007 to 2024.

Age group	Positive (Observed)	Positive (Expected)	Chi-square Contribution
Infants (0 years)	125	397.6	**204.8**
Infancy (1–5 years)	1636	777.8	**1037.8**
Kindergarten age (6–8 years)	95	85.4	**1.4**
Elementary school age (9–12 years)	49	59.1	**1.9**
Adolescents (13–18 years)	25	50.5	**14**
Adults (19–64 years)	61	256.9	**163.7**
Older adults (≥ 65 years)	47	410.7	**352.9**
